# The Interplay of Biotic and Abiotic Factors in Shaping Genetic Variation at *6Pgdh* in the Bulb Mite

**DOI:** 10.1002/ece3.72671

**Published:** 2025-12-19

**Authors:** Pranav Unnikrishnan, Anna Spaeth, Magdalena Trojańska, Wiesław Babik, Agata Plesnar‐Bielak

**Affiliations:** ^1^ Institute of Environmental Sciences, Faculty of Biology Jagiellonian University Kraków Poland; ^2^ Institute of Microbiology, Department of Pathobiology University of Veterinary Medicine Vienna Austria

**Keywords:** environment‐driven selection, genetic polymorphism, genotype by environment interaction for fitness, sexual selection, temperature

## Abstract

The polymorphism of *6Pgdh* in the bulb mite *Rhizoglyphus robini* involves two alleles, S and F, which have opposing effects on male reproductive success. While S‐bearing males have a reproductive advantage and the S allele fixes in the laboratory populations, polymorphism is regularly observed in natural populations. We hypothesize that *6Pgdh* polymorphism in the bulb mite may be maintained by context‐dependent selection, driven by fluctuations of temperature and sexual selection intensity, as predicted by balancing selection models based on genotype‐by‐environment interactions. This study uses experimental evolution to track allele frequency changes over 13 generations under different temperatures (12°C and 18°C) and sexual selection intensities (equal and female‐biased sex ratios). The results show a significant increase in F allele frequency under higher sexual selection intensity at 12°C. This suggests that individuals with the F allele gain a reproductive advantage at lower temperatures, and *6Pgdh* polymorphism may be maintained through a three‐way interaction for fitness between genotype, abiotic environment (temperature), and biotic environment (sex ratio). In addition, in a direct assay of male reproductive success across temperatures, we did not detect differences between genotypes. This suggests that the allele frequency shifts observed in the experimental evolution may not be explained solely by reproductive competition, but could also involve selection acting on survival or other fitness components. The complex interplay between environmental factors and demographic dynamics in shaping allele frequencies highlights that multiple factors can interact in maintaining polymorphism, potentially through context‐dependent selection.

## Graphical abstract

We used experimental evolution to track changes in 6Pgdh allele frequencies in bulb mites over 13 generations under different temperatures and sexual selection intensities. Our results show that the F allele increases in frequency only at low temperatures under strong sexual selection, indicating that the maintenance of genetic variation in this system depends on a complex interplay between genotype, abiotic, and biotic factors. This study highlights the importance of multi‐factorial interactions in shaping genetic diversity.

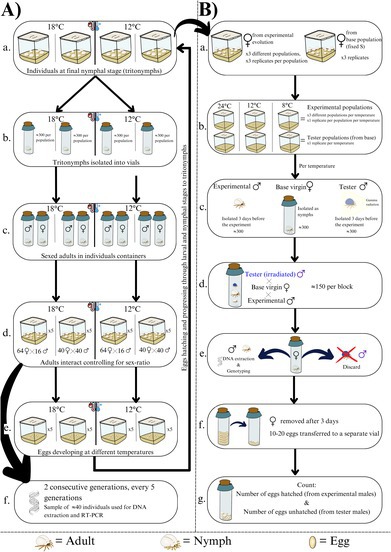

## 1 Introduction

Genetic variation, which can be considered the fundamental raw material for natural selection, is a crucial factor for evolutionary processes to manifest. An increase in genetic variation within a population increases the potential for evolutionary change, as postulated by Fisher; “the rate of increase in the average fitness of a population is equal to the genetic variance of fitness of that population” (Fisher 1941). Such genetic variation, which can result in diverse phenotypes, allows populations to adapt to environmental challenges (Barrett and Schluter 2008). Nevertheless, directional selection tends to diminish genetic variation by selecting for particular variants, thereby increasing their frequency within a population (Bürger and Lynch 1995). This often results in the eventual fixation of the favored variant (Wright 1932; Endler 1986). Therefore, the persistence of genetic polymorphism, despite the expectation of decreased variation under directional selection, presents a paradox (Lewontin 1974; Radwan et al. 2016; Hallsson and Björklund 2012). This persistence of genetic polymorphisms suggests that complex selective forces may act to maintain genetic diversity. In some cases, interactions between genetic, abiotic, and biotic factors could generate context‐dependent selection pressures, resulting in a three‐way interaction for fitness that prevents allele fixation and preserves genetic variation.

Selection processes that result in the maintenance of polymorphisms fall under a common umbrella term: balancing selection. Balancing selection maintains genetic polymorphisms through various mechanisms (Stamp and Hadfield 2020; Fijarczyk and Babik 2015), including heterozygote advantage, where heterozygous individuals have higher fitness (Manjurano et al. 2015); negative frequency‐dependent selection, which favors rare alleles (Fitzpatrick et al. 2007); and antagonistic pleiotropy, involving trade‐offs between fitness‐related traits (Nuzhdin et al. 1997). Another key mechanism contributing to balancing selection is environmental heterogeneity, which promotes genetic diversity by creating shifting selection pressures across space and time (Hedrick et al. 1976; Slatkin 1987; Kokko and Heubel 2008). While early theoretical models suggested that stable polymorphism required restrictive conditions (Via and Lande 1987; Gillespie and Turelli 1989; Levins 1968), newer models incorporating polygenic traits, fluctuating environments, and complex selection dynamics reveal a broader potential for maintaining genetic variation (Svardal et al. 2015; Xue et al. 2019). Dobzhansky's (1943) pioneering study on seasonal allele frequency changes in *Drosophila* provided early empirical evidence for how environmental variation can sustain genetic diversity, a finding later supported by research across taxa (Brook 2009; van Dongen et al. 2016; Zan and Carlborg 2020).

As mentioned above, the role of environmental heterogeneity in driving selection in natural systems can be complex. The potential of a given environmental factor to shape genotype‐by‐environment interactions for fitness may depend on another environmental variable. For example, several studies have demonstrated genotype‐by‐environment interactions for sexual fitness, including male reproductive success (Patlar and Ramm 2020; Greenfield and Rodríguez 2004; Ingleby et al. 2010). The potential of such interactions to drive selection should vary with conditions that influence the strength of sexual selection, such as population density or sex ratio. Consequently, one might observe a three‐way interaction for fitness between genotype, abiotic environment, and biotic environment, contributing to the maintenance of polymorphism.

The bulb mite (*Rhizoglyphus robini*) is an acarid mite commonly found in soil and decaying organic matter. The species is well‐known for its use as a model system in studies of reproductive strategies and sexual selection (Radwan 1995; Smallegange 2011; Tomkins et al. 2011; Plesnar‐Bielak et al. 2018) due to the occurrence of alternative male reproductive phenotypes: fighters and scramblers (Radwan and Klimas 2001). These phenotypes exhibit differences in morphology and behavior that influence reproductive success. Female‐biased sex ratios are commonly observed in natural bulb mite populations, while male‐biased populations are infrequent (Deere et al. 2015). Similarly, laboratory populations maintained without sex ratio manipulations tend to stabilize at approximately 60:40 female‐to‐male ratios (personal observations by M. Jarzębowska, A. Skwierzyńska, A. Plesnar‐Bielak). The bulb mite's short generation time and ease of maintenance in laboratory settings make it a valuable system for experimental evolution studies.

The *6Pgdh* gene encodes 6‐phosphogluconate dehydrogenase, a key enzyme in the Pentose Phosphate Pathway (PPP), which plays a crucial role in energy metabolism, oxidative stress response, and the synthesis of biomolecules such as nucleotides and fatty acids (Ge et al. 2020; Tambasco‐Studart et al. 2005; Jakkula et al. 2021). The PPP is influenced by environmental conditions such as temperature and salinity (Fahrendorf et al. 1995; Hou et al. 2007), and heterogeneity in these factors has been linked to patterns of polymorphism in *6Pgdh* across taxa (González‐Ruiz et al. 2023; Landi et al. 2021). In *R. robini*, the *6Pgdh* gene encodes two protein variants (alleles S and F), which differ by a single amino acid substitution (Skwierzyńska and Plesnar‐Bielak 2018). The alleles (originally named “slow” and “fast”) influence male reproductive success (Konior et al. 2006). Importantly, to avoid confusion, we would like to emphasize that the S and F *6Pgdh* alleles are not related to the alternative male reproductive phenotypes (fighters and scramblers). The S allele is associated with greater male reproductive success due to higher sperm production and copulation frequency (at standard laboratory conditions) (Skwierzyńska and Plesnar‐Bielak 2018), but this advantage comes with a reduction in female partner fitness through reduced fecundity, the mechanisms of which remain unclear (Konior et al. 2006). While the S allele rapidly fixes in laboratory conditions, both alleles are often maintained in natural populations (Unnikrishnan et al. 2024), suggesting an active role for environmental factors in maintaining genetic diversity. By focusing on the *6Pgdh* gene in *R. robini*, our study bridges the gap between traditional phenotypic studies of reproductive strategies, such as investigations of male morphs and mating behavior, and genetic‐level analyses of allele frequency changes in response to environmental and demographic factors, offering new insights into how complex selection pressures operate in dynamic environments.

A previous experimental evolution study showed that the selective advantage of the S allele is reduced (but not reversed) in populations evolving at a temperature of 18°C compared to the standard laboratory temperature of 24°C (Plesnar‐Bielak et al. 2020). Similarly, the study also showed that the S allele frequency increased less rapidly in populations where sex ratio manipulation relaxed the intensity of sexual selection (Plesnar‐Bielak et al. 2020). As such, it is possible that these two factors, if applied simultaneously, could lead to a reversal of fitness rank and a selective advantage for the F allele. In such a case, the interaction of temperature and sex ratio dynamics (which influence sexual selection intensity) could explain the maintenance of the *6Pgdh* polymorphism in the wild.

Here, we use experimental evolution to test how the interaction of temperature and sexual selection intensity shapes *6Pgdh* allele frequencies. We track changes in *6Pgdh* frequencies in replicate populations for 13 generations under two levels of temperature (18°C and 12°C) interacting with two levels of sexual selection intensity (equal sex ratio and female‐biased population with relaxed sexual selection). Based on previous results (Plesnar‐Bielak et al. 2020), we predict that the S allele's benefit is diminished at lower temperatures, potentially due to increased survival costs. If this cost is associated with survival (as opposed to reproductive fitness), we predict an increase in F‐allele frequency under lower temperature and relaxed sexual selection. This is because a female‐biased sex ratio reduces male–male competition, thereby weakening the strength of sexual selection on males and the reproductive advantage usually enjoyed by S‐bearing males. As a result, any survival cost associated with the S allele at lower temperatures would have a greater influence on allele frequency change under these conditions. Alternatively, if the cost is associated with reproductive fitness rather than survival, the reproductive advantage of the S allele itself might be temperature dependent. Under such a scenario, the F allele might gain reproductive advantage at low temperature, and we would expect an increase in its frequency under low temperature and intense sexual selection. Consistent with the latter prediction, we found that F allele frequency increased specifically under strong sexual selection at 12°C, indicating a temperature‐by‐sexual selection interaction that may contribute to the maintenance of the *6Pgdh* polymorphism.

## 2 Materials and Methods

### 2.1 Study System

6‐Phosphogluconate dehydrogenase (*6Pgdh*) is a key enzyme of the pentose phosphate pathway, which together with glycolysis forms a major route of glucose catabolism conserved across animals, plants, and fungi (Goulielmos et al. 2004; Hanau and Helliwell 2022). This pathway generates ribose‐5‐phosphate sugars and NADPH, essential for biosynthesis and cellular redox balance (Stincone et al. 2015).

In *R. robini*, a single nonsynonymous substitution in *6Pgdh* (arginine–methionine) creates two common alleles, S and F (Konior et al. 2006; Skwierzyńska and Plesnar‐Bielak 2018). This polymorphism is associated with male reproductive fitness, representing a case of sexual selection: males that are homozygous for the S allele achieve higher mating success and sperm production than homozygous F males, while heterozygotes show intermediate values (Łukasik et al. 2010; Skwierzyńska and Plesnar‐Bielak 2018). Female fitness is unaffected by *6Pgdh* genotype, but females mated with SS males produce fewer eggs than those mated with FF males, which creates sexual conflict (Konior et al. 2006; Łukasik et al. 2010; Skwierzyńska and Plesnar‐Bielak 2018). Consistent with its advantage under sexual selection, the S allele tends to fix rapidly in laboratory populations. In contrast, natural populations frequently retain both alleles, with polymorphism levels varying across space and time (Unnikrishnan et al. 2024).

### 2.2 DNA Extraction and *6Pgdh* Genotyping

DNA was extracted from individual mites using a chelex‐based protocol. Each mite was crushed in 40 μL of 1% Chelex solution, and the samples were incubated in a thermocycler (Thermofisher Scientific) at 94°C for 10 min and then heated to 75°C for 15 min (based on modified protocol from Konakandla et al. 2006). Extracts were stored at ~6°C until genotyping.

*6Pgdh* genotypes were determined using real‐time PCR with fluorogenic TaqMan SNP genotyping (ThermoFisher Scientific) targeting the Arg‐to‐Met missense SNP distinguishing the S and F alleles (see Unnikrishnan et al. 2024). Genotyping was performed on a Bio‐Rad CFX96 Real‐Time PCR detection system. We used TaqMan Genotyping Master Mix together with a Custom TaqMan SNP genotyping assay specific to the *6Pgdh* S/F polymorphism. This genotyping assay was designed and quality controlled by ThermoFisher based on the *6Pgdh* sequence fragment we supplied; the sequence context used for assay design, along with full primer and probe sequences and dye/quencher information, is provided in the Appendix A. The assay comprises two flanking primers and two allele‐specific fluorogenic minor‐groove‐binder probes labeled with FAM and VIC, following the standard design of Custom TaqMan SNP assays.

Reactions were run in 96‐well plates in 10 μL volumes. Each reaction contained 5.5 μL of TaqMan Genotyping Master Mix combined with the Custom TaqMan SNP assay (following the manufacturer's recommended proportions; equivalent to the 10:1 mix used in the original protocol) and 4.5 μL of chelex‐extracted genomic DNA. PCR cycling followed the original protocol of 41 cycles consisting of 95°C for 15 s and 60°C for 1 min, preceded by an initial denaturation at 95°C for 10 min. Allelic discrimination was performed automatically after amplification.

### 2.3 Source Population

Experimental evolution populations were established from a *6Pgdh* polymorphic population (source population), in which the frequency of the F allele had been increased before the start of the experiment. It was done in order to start experimental evolution from approximately equal frequencies of both alleles. To do it, we used a field‐collected population originating from > 100 individuals found on onions collected in Łazany (50.0206 N, 19.8924 E), near Kraków in July 2020. This field‐collected population was kept at 24°C for 1 week to allow juveniles to mature and reproduce, then transferred to 8°C to slow development and minimize population expansion before experimental setup.

The source population was created in spring 2021. We randomly paired virgin females and males from the field‐collected population, in which the F allele frequency was about 0.23. After the pairs mated (almost 300 pairs) and females laid eggs, both parents were genotyped. We then looked for pairs with at least two copies of the F allele (either both parents FS, or one FF and one SS, or one FF and one SF, or both parents FF). The offspring from such pairs were used to establish the source population. The population was allowed to expand freely for ca. 2 months at 24°C (which corresponds to 3–4 mite generations), before it was moved to 12°C to extend generation time, to prevent adaptation to 24°C and the loss of the F allele. This population was used to start the experimental evolution populations within 1 month after transferring to 12°C (less than one mite generation at that temperature). The F‐allele frequency in the source population was 0.6 when the experimental evolution started.

### 2.4 Culturing Conditions

All populations (source and experimental) were maintained in plastic containers (approximately 2.5 cm in diameter) with a plaster of Paris base soaked with water. They were maintained at > 90% humidity, constant darkness, with powdered yeast provided ad libitum as a food source. Individually isolated mites were maintained in glass tubes with a diameter of approximately 1 cm.

### 2.5 Experimental Evolution

**FIGURE 1:**
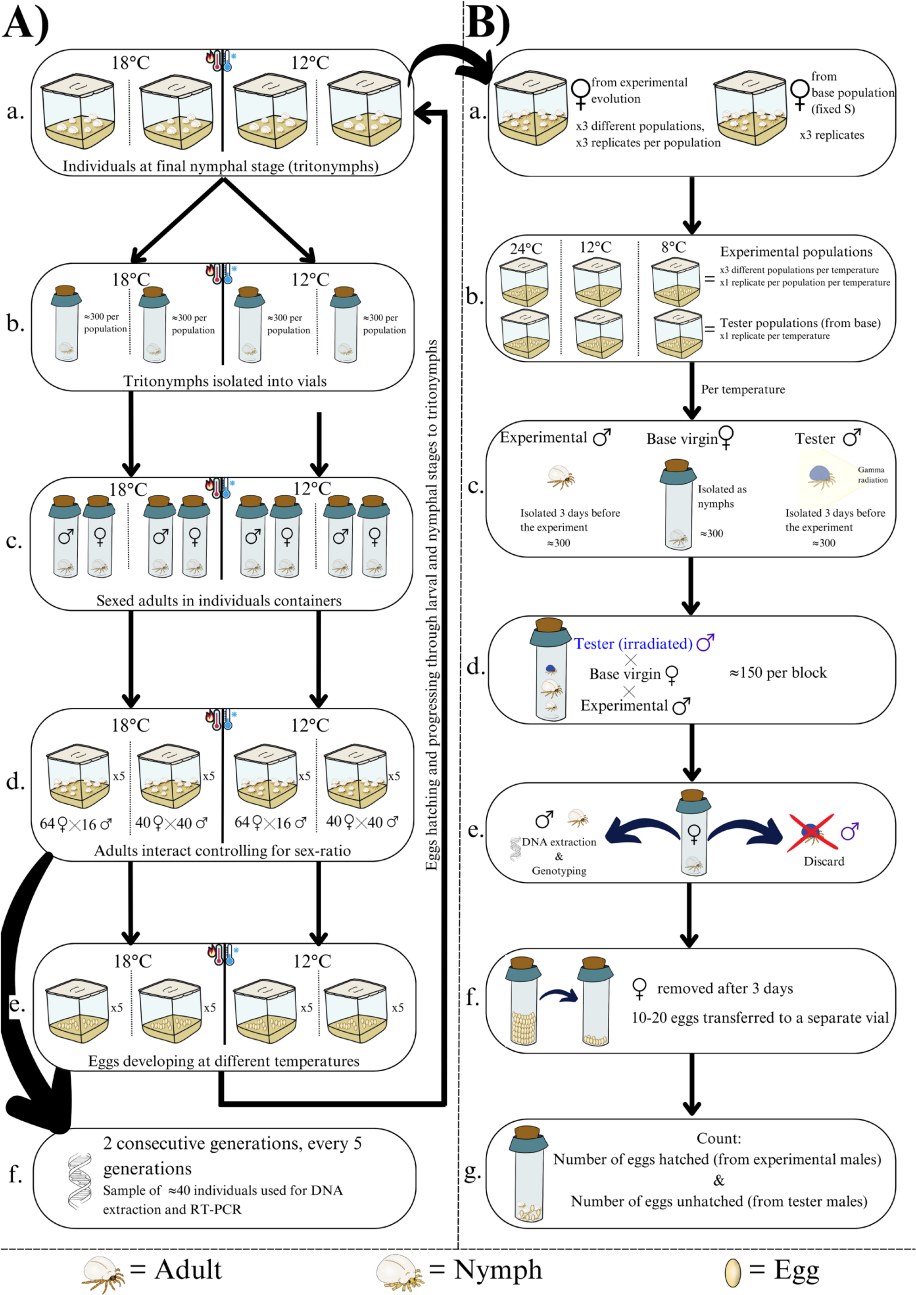
Schematic overview of: (a) Experimental evolution. Twenty replicate populations were assigned to a 2 × 2 design with two temperatures (12°C, 18°C) and two sex ratios (equal = 40♀:40♂, female‐biased = 64♀:16♂), each with five replicates. Individuals were isolated as tritonymphs, sexed as adults, and combined in containers for six days of free mating before adult removal. Allele frequencies of *6Pgdh* were sampled every two generations, with a five‐generation gap (generations 0, 1, 2, 7, 8, 12, 13). (b) Reproductive success assay. Males from three populations evolved at 12°C under equal sex ratios were competed against irradiated SS males from a base population fixed for the S allele, using virgin females from the same base. One experimental male, one tester male (dyed blue), and one virgin female were kept together for 3 days at 8°C, 12°C, or 24°C. Male genotype was determined, and hatching success of 10–20 eggs per vial was used to estimate experimental male reproductive success. Two blocks were run two generations apart.

To test how temperature and sexual selection intensity affect allele frequency dynamics, we used experimental evolution with two temperatures (12°C and 18°C) crossed with two adult sex‐ratio treatments (equal vs. female‐biased; Figure 1A). Manipulation of adult sex ratio is a well‐established method for altering sexual selection intensity (Kvarnemo and Ahnesjö 1996; Fitze and Le Galliard 2008; Weir et al. 2011). Equal sex ratios (40 females + 40 males) were used to generate strong sexual selection on males, since competition for mates is intense when males and females are equally abundant. In contrast, female‐biased ratios (64 females + 16 males; male proportion = 0.2) reduce male–male competition and thus represent weaker sexual selection. The female‐biased ratio was also chosen because it reflects conditions commonly observed in natural populations (Deere et al. 2015) and in the laboratory (personal observations), while male‐biased ratios were excluded because of practical constraints in obtaining a sufficient number of males. The temperature of 18°C corresponds to the “low” treatment used in a study by Plesnar‐Bielak et al. (2020), while 12°C was used to decrease the temperature below the range investigated before. It approximates the mean annual temperature in Poland (although natural habitats experience wider fluctuations from −5°C in winter to > 25°C in summer; www.worlddata.info). Each treatment combination was replicated five times, yielding 20 experimental populations in total.

For each generation, individuals were isolated at the final nymphal stage (tritonymphs) into separate vials, where they developed into adults (Figure 1A‐a–c). Adult virgin mites were then put in common containers (one container per replicate; controlling for the sex ratio), where they were allowed to interact for 6 days at their specific temperature treatment, during which they could mate freely, and females could lay eggs (Figure 1A‐d). Adults were then removed and eggs left to develop (Figure 1A‐e). Allele frequencies at the *6Pgdh* locus were estimated from ~40 individuals per replicate at generations 0, 1, 2, 7, 8, 12, and 13 (Figure 1A‐f).

### 2.6 Reproductive Success

We measured the competitive reproductive success of males with different *6Pgdh* genotypes against SS‐homozygous tester males from the stock population, using the sterile male technique (Parker 1970; Radwan 1991; Radwan and Siva‐Jothy 1996; Figure 1B). Tester males were sterilized with 20 kRad (200 Gy) gamma radiation from Co60, which allows fertilization but prevents egg hatching (Radwan and Siva‐Jothy 1996). Because females do not lay unfertilized eggs, the proportion of hatched eggs provides a direct measure of the experimental male's reproductive success.

Experimental males were obtained from three replicate populations evolved under 12°C with equal sex ratios, which showed the highest F‐allele frequency. Females and tester males were sampled from the base population maintained at 24°C, fixed for the S allele (Figure 1B‐a,b). Virgin females were generated by isolating ~600 tritonymphs from the base population. Prior to trials, experimental and tester males were isolated for 3 days; tester males were given food that contained blue dye for identification (Tilszer et al. 2006; Plesnar‐Bielak et al. 2013). All tester males were irradiated together under identical conditions and then randomly assigned to trials, ensuring that any reduction in competitiveness due to irradiation affected all treatments equally (Figure 1B‐c).

Each trial included one experimental male, one irradiated tester male, and one virgin female (Figure 1B‐d). After 3 days, tester males were removed, experimental males were genotyped, and females were left 2–4 more days to oviposit (Figure 1B‐e). From each vial, 10–20 eggs were sampled, and the proportion that hatched was recorded as the experimental male's reproductive success (Figure 1B‐f,g). The assay was conducted at 8°C, 12°C, and 24°C in two blocks, each including males from all three populations; the source populations differed by two generations between blocks, but methods were identical.

### 2.7 Statistical Analysis

All analyses were conducted in R v4.3.0 (R Core Team 2021). Data visualization was performed using ggplot2 (v3.4.4). To test how the *6Pgdh* allele frequencies were changing across generations depending on the selection treatment, we used the generalized estimating equations (GEE) approach using the geeglm function (geepack, version 1.3.10). The response variable was the vector of S and F allele counts (per population obtained from each generation tested), analyzed with a binomial error distribution and logit link. Predictors were the temperature of evolution, sexual selection intensity, and their interaction. The replicate population was specified as the clustering unit (id = Population), generation as the repeated measure (waves = Generation), and an AR1 correlation structure was applied. To test for significant allele frequency changes per treatment, we calculated the difference in F‐allele frequency between generation 13 and generation 0 for each population and compared these values against zero using two‐tailed one‐sample *t*‐tests.

Reproductive success was analyzed with a generalized linear mixed‐effects model (GLMM) using glmmTMB (v1.1.9). The response variable was a vector containing the number of hatched (successes) and unhatched (failures) eggs per trial. Male genotype, temperature, and their interaction as well as block were included as fixed effects, and the source population of experimental males as a random effect. A beta‐binomial distribution was used to account for overdispersion.

## 3 Results

### 3.1 Experimental Evolution

**FIGURE 2:**
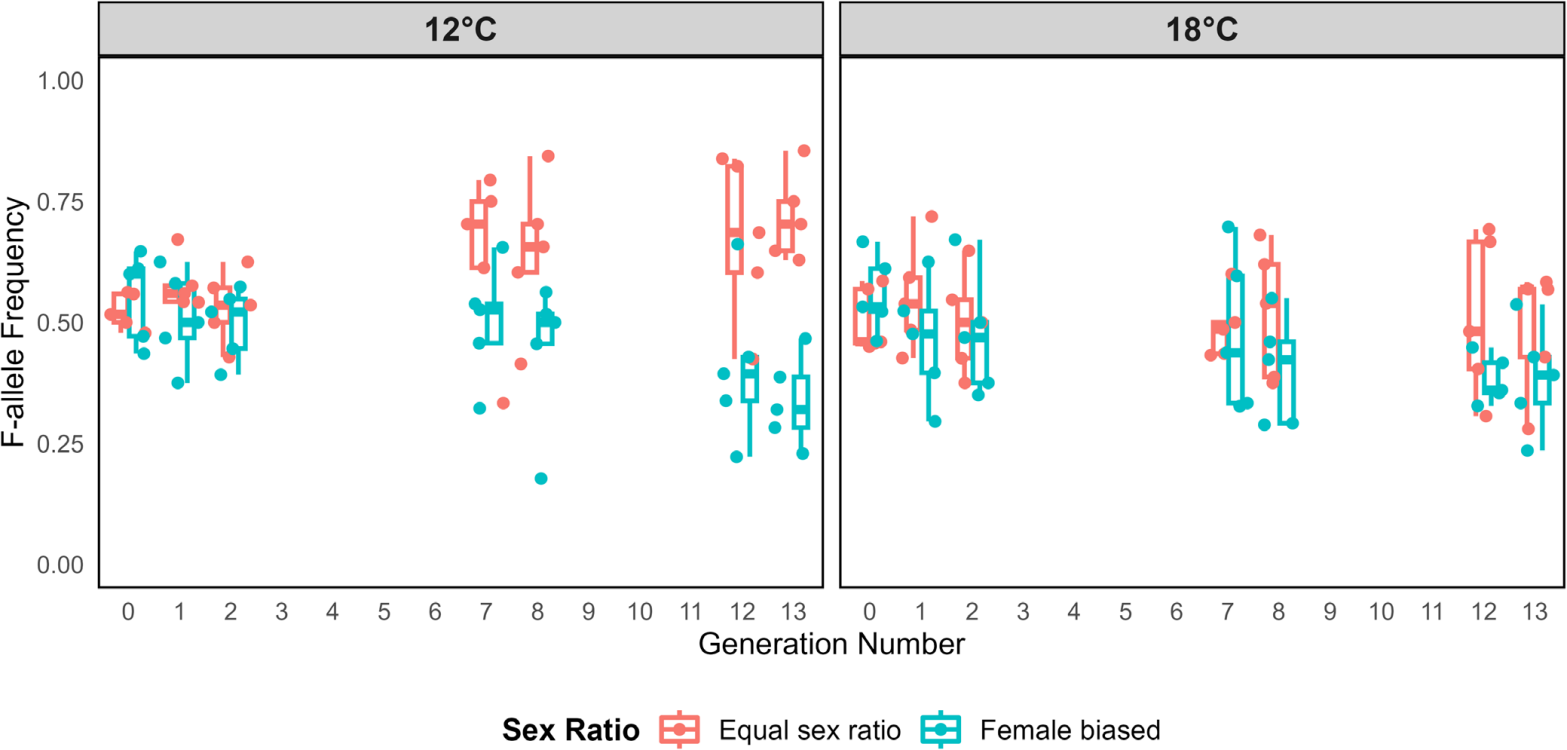
Evolution of F‐allele frequency across 13 generations in different environmental treatments. The colors represent the sex‐ratio levels (equal vs. female‐biased), corresponding to differences in sexual selection intensity (strong vs. weak). Boxplots show the interquartile range (IQR) of F‐allele frequencies for each treatment group in each generation, with the center line representing the median.

The effect of sexual selection intensity on *6Pgdh* allele frequency dynamics depended on temperature, as shown by a significant interaction between the two factors (GEE: *χ* ^2^ = 4.58, *p* = 0.03) (Figure 2). In the model, the main effect of sexual selection intensity was significant (*χ* ^2^ = 9.56, *p* = 0.0019), whereas the main effect of temperature was not (*χ* ^2^ = 1.51, *p* = 0.22). Thus, allele frequency change was shaped by sexual selection in a temperature‐dependent manner. To further explore this interaction, we analyzed the effects of sexual selection intensity on allele frequencies at each temperature separately.

At 12°C, sexual selection intensity significantly affected allele frequency change (GEE: *χ* ^2^ = 8.92, *p* = 0.0028). Specifically, under strong sexual selection (equal sex ratio), the F allele increased from 0.52 ± 0.02 at generation 0 to 0.72 ± 0.04 at generation 13, corresponding to a 37% increase (Figure 2). This increase was significant (*t*_4_ = 5.06, *p* = 0.007). All five replicate populations showed the same directional change, with frequencies rising steadily across generations. By contrast, under weaker sexual selection (female‐biased sex ratio), mean frequency declined from 0.55 ± 0.04 to 0.34 ± 0.04 (a 39% decrease). This change was not significant (*t*_4_ = −0.47, *p* = 0.66), and replicate trajectories were more variable, with four populations decreasing and one increasing (Figure 2).

At 18°C, sexual selection intensity had no significant effect on allele frequency changes (GEE: *χ* ^2^ = 0.51, *p* = 0.47). Specifically, under strong sexual selection, F allele frequency remained close to its starting value (0.50 ± 0.03 at generation 0 to 0.49 ± 0.06 at generation 13; *t*_4_ = −0.83, *p* = 0.45). Under weaker sexual selection, F frequency declined from 0.56 ± 0.04 to 0.39 ± 0.05, but this was also not significant (*t*_4_ = 1.97, *p* = 0.12) (Figure 2).

Overall, only under the combined conditions of low temperature and strong sexual selection did the F allele consistently increase in frequency, indicating a combined effect of both factors.

### 3.2 Reproductive Success

**FIGURE 3:**
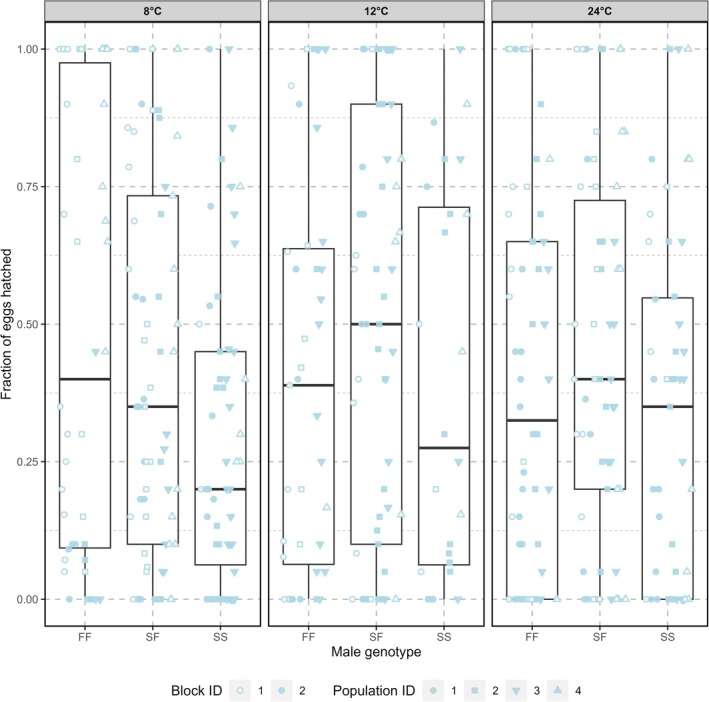
Comparison of reproductive success of different genotypes in different temperatures, the *y*‐axis shows the fractions of eggs that hatched which is the representation of the level of reproductive success. The boxplots represent the IQR of the fraction of eggs hatched (reproductive success) with the center line being the median, and the points represent the success of individual males. The shapes of the individual points represent the ID of the experimental evolution population used and the fill of the points represent the block ID.

Across temperatures, and in competition with a sterile tester male, mean male reproductive success (inferred from the proportion of a female partner's subsequent eggs that hatched) ranged from 30% to 45%, with no genotype showing a consistent advantage across temperatures (Figure 3). The interaction between temperature and male genotype had no significant effect on male reproductive success (GLMM: *χ* ^2^ = 2.86, *p* = 0.58, Figure 3), indicating that the reproductive success of males with different genotypes did not differ at different temperatures.

Temperature and male genotype did not have significant individual effects on male reproductive success (GLMM: *χ* ^2^ = 2.16, *p* = 0.34; *χ* ^2^ = 3.60, *p* = 0.17, respectively). The only predictor variable that was significant was block ID (GLMM: *χ* ^2^ = 6.4323, *p* = 0.011). However, the lack of significant genotype by temperature interaction on male reproductive success was consistent between blocks.

## 4 Discussion

In systems involving environment‐dependent selection, there may be cases where interactions between biotic and abiotic environments contribute to the maintenance of polymorphism (Hedrick et al. 1976; Ferrer‐Admetlla et al. 2008). Such a situation may occur in the bulb mite metabolic gene *6Pgdh*. Although males with the S allele show higher reproductive fitness in laboratory populations, polymorphism persists in natural populations (Unnikrishnan et al. 2024). The aim of this study was to investigate, under controlled laboratory conditions, whether environmental factors—temperature and intensity of sexual selection manipulated by altering sex ratios—act alone or in combination to drive the patterns of polymorphism.

Our findings suggest that fitness in *R. robini* is shaped by a complex interplay between genotype, environmental factors (specifically temperature), and social factors (specifically sex ratio). Furthermore, although there have been several other studies of the factors contributing to environment‐driven selection, many of these have focused on the separate effects of individual factors (Carley et al. 2021; Smith et al. 2021; Lonn et al. 2017). A multifaceted approach to the study of the maintenance of polymorphism has rarely been used. A recent study on seed predation in an African savannah small mammal community system (Schoepf and Pillay 2023) is a notable exception. It shows that multiple factors such as seed size, seed type, predation risk, habitat type, seasonality, and their interactions drive seed variation, with the possibility that these factors are likely to be hierarchically ordered. Similarly, a study by Joswig et al. (2022) showed how the interaction of climate and soil fertility influences global plant variation. Our study adds to these by highlighting the role of social parameters—specifically, sex ratio variation—in considering such interactions within species.

Our findings further suggest that the fitness advantage of the S allele is not absolute but context dependent. The observed increase in F allele frequency under low temperature and intense sexual selection suggests that low temperature reverses allele ranks in reproductive fitness, potentially shifting the advantage from S allele to F allele males under these conditions. This highlights the potential for complex trade‐offs, where the F males may gain a reproductive advantage at colder temperatures, or the S males may have superior reproductive performance in warmer environments but face higher mortality risks under intense sexual selection at lower temperatures. The reproductive advantage of the F allele at low temperature was, however, not confirmed by the reproductive success assay using direct male–male competition at different temperatures. It could be the case that the conditions of the experiment may not have been optimal for the reproductive advantage of the F males to be expressed. This advantage might have been observed under conditions with more competitors or a longer competition duration.

In addition, the design of the experiment introduces substantial variation due to factors such as differences in egg‐laying ability of the females (less than 10 eggs from some females to more than 200 eggs from some females) or due to the non‐uniform nature of the irradiation effects on the males. This high level of variation indicates that the current experiment lacks sufficient statistical power to detect moderate but still biologically relevant differences. There also were significant differences between blocks which likely reflect uncontrolled variation in experimental conditions rather than inherent differences between the source populations. Such block effects are common in biological experiments and suggest that subtle environmental or handling differences between experimental runs may have influenced the results. On the other hand, the sterile male technique has been used previously in this (Radwan and Siva‐Jothy 1996; Plesnar‐Bielak et al. 2018; Skwierzyńska and Plesnar‐Bielak 2018) and other systems (Sirot et al. 2007; Mazza et al. 2015; Magris et al. 2015) and allowed the detection of significant effects.

If the F males do not gain a reproductive advantage at lower temperatures, this suggests that viability selection, rather than reproductive competition, may be the primary driver of allele frequency shifts. In this case, selection would act before the reproductive stage, favoring F males through improved survival at lower temperatures. If this were true, we would expect a main effect of temperature rather than an interaction with sexual selection intensity. Instead, the increase in F allele frequency was restricted to the strong sexual selection treatment, suggesting that survival differences alone do not account for the observed pattern. Alternatively, if a survival‐reproduction trade‐off were responsible—where S males perform better reproductively but suffer higher mortality under stressful lower temperature—then we might expect the F allele to increase under weak sexual selection at 12°C and not at strong sexual selection intensity, which again was not observed. Consequently, survival selection is unlikely to explain the persistence of both alleles.

The increase in F allele frequency under strong sexual selection at 12°C suggests that sexual competition may interact with environmental conditions. One possibility is that at low temperatures, S males experience greater physiological stress under intense competition, perhaps because lower temperatures reduce the efficiency of key metabolic processes, such as the PPP (e.g., by slowing reaction rates) thereby limiting their ability to sustain reproductive behaviors. This could reduce their survival or overall fitness under strong sexual selection at low temperature—a context‐dependent survival–reproduction trade‐off—leading to a relative increase in F allele frequency. However, under weak sexual selection, this stress may be lower, preventing a survival disadvantage for S males and limiting the increase in F allele frequency.

While laboratory conditions inherently simplify ecological dynamics, they provide a valuable approximation of natural selection pressures. In the wild, *R. robini* experience seasonal temperature changes and fluctuations in population sex ratio and density. These environmental dynamics could plausibly maintain genetic polymorphism through mechanisms similar to those observed in our experiments. For instance, seasonal fluctuations in soil temperature may create periodic changes in selection pressures, with colder conditions favoring different alleles during specific seasons. In regions such as Poland, where seasonal temperatures can range well below 12°C during winter and exceed 18°C during summer, the magnitude of environmental fluctuations is likely much greater than what was tested in our laboratory setup. This could amplify the strength of selection‐driven allele shifts across seasons. However, a recent field study found that temperature alone cannot explain polymorphism patterns on *6Pgdh* in this species which showed no consistent changes in *6Pgdh* allele frequencies across seasons (Unnikrishnan et al. 2024). In addition to temperature fluctuations, sexual selection intensity can also vary considerably in natural settings. In wild bulb mite populations, this variation is influenced not only by fluctuations in sex ratio but also by differences in population density, which can affect the strength of male–male competition. Based on our experimental findings, such fluctuations in environmental conditions and sexual selection intensity may periodically shift reproductive success between S‐ and F‐allele males. Over time, these cycles could maintain polymorphism by preventing allele fixation through context‐dependent balancing selection. However, further work is needed to explore survival outcomes, environmental persistence, and the broader ecological relevance of these findings in natural populations, especially as genomic scans for long‐term balancing selection have not detected its signatures in proximity of the *6Pgdh* gene (Zaborowska, J., Plesnar‐Bielak, A., Babik, W., unpublished).

Different factors interacting with each other have been shown to influence adaptation rates, which may be linked to the impact of these factors on genetic variation and polymorphisms in important genes. The importance of considering multiple ecological and demographic factors, and their interactions, in the study of genetic variance during experimental evolution has been recognized and highlighted (Van den Van Bergh et al. 2018). An experimental evolution study of the bruchid beetle ( *Callosobruchus maculatus* ) investigated the effect of varying natural (food type) and sexual (mating system) selection intensities on the rate of adaptation (Fricke and Arnqvist 2007). The study showed that mating system treatment (biotic factor) influenced the rate of adaptation, depending on food type (abiotic factor). In *Drosophila serrata*, an experimental evolutionary study showed that the effect of natural selection on the rate of adaptation to novel environments was greater in a monogamous mating system (Rundle et al. 2006). In contrast, in an experimental evolutionary study of *R. robini*, polygamous populations adapted rapidly to changes in thermal environments, whereas monogamous populations suffered fitness declines and were more likely to go extinct in novel environments (Plesnar‐Bielak et al. 2013). These studies, together with our results, emphasize the need to consider methods to incorporate multi‐factorial interactions (genotype × biotic × abiotic) in laboratory studies such as experimental evolution in order to obtain meaningful results and ultimately unlock the answers to many evolutionary processes. Furthermore, although such experimental systems and designs could become considerably more complex due to the addition of multiple factors and their interactions, inference could be refined by focusing on individual and key metabolic genes such as those involved in energy production, oxidative stress response and biosynthesis, as demonstrated in our study.

By focusing on a key metabolic gene, our study provides insights into how environmental factors and sex ratio interact to influence allele frequency dynamics. Variation in metabolic genes can significantly affect fitness and the long‐term persistence of populations (Koshiba et al. 2020; Whitt et al. 2002). Examining *6Pgdh* polymorphism offers broader insights into how genetic variation may be maintained through interactions between genotype, abiotic conditions, and social factors such as sex ratio. Such interactions are likely to play an important role in shaping genetic diversity across a range of natural systems. Functional effects of metabolic genes like *6Pgdh* have been well‐documented in various taxa, including plants, invertebrates, and vertebrates, where they are linked to stress resistance, metabolic efficiency, and survival under challenging environmental conditions (Zhao et al. 2020; Tian et al. 1998). Our results suggest that monitoring factors related to environment and aspects of population structure, such as sex ratio, may be a key to predicting the fate of many populations, particularly when populations face changing environments or anthropogenic pressures (Parrett and Knell 2018; Peniston et al. 2020).

While our study provides knowledge and insight into the system, it is important to recognize and address its limitations. Although populations were maintained at constant census size (N) and sex ratio was controlled, the effective population size (Ne) may have varied between the populations with female‐biased and equal sex‐ratio. The potential differences in effective population size (Ne) between treatments might have slightly influenced allele frequency changes. This discrepancy in Ne has been identified and discussed as a potential limitation in previous experimental evolutionary studies that manipulate sex ratio (LaMunyon et al. 2007; Wigby and Chapman 2004). However, experimental evolution studies in other species have suggested that such differences in Ne between experimental populations should not considerably bias the results (Snook et al. 2009; Edward et al. 2010; Rice and Holland 2005). In addition, recent results on *R. robini* suggest that Ne did not differ significantly between populations with much higher differences in sex ratio than the ones used here maintained for 28 generations (male to female ratio 3:1 and 1:3; Chmielewski, S., Konczal, M., Parrett, J., Radwan, J., unpublished). Furthermore, Parrett et al. (2022) estimated Ne in their experimental evolution populations of *R. robini* and found that it remained relatively similar across treatments, supporting the interpretation that sex ratio biases may have only minor effects on Ne in this system. This suggests that differences in Ne between populations are minor, thereby making the comparison between our experimental populations valid. Furthermore, although our results suggest that the F allele is favored in the long term in the low temperature/high sexual selection intensity treatments, evolutionary timescales are much longer compared to a 13‐generation span. Hence, it would be interesting to experimentally assess the stability of these interactions over longer timescales. Finally, although we have gained insight into the maintenance of polymorphism, we have yet to determine the precise advantage gained by individuals with the F allele at lower temperatures. Addressing these limitations in future research will enhance our understanding of the intricate interplay between genetic, environmental, and demographic factors—such as sex ratio—in shaping allele frequency change and the maintenance of genetic variation.

Overall, our study highlights the potential three‐way interaction for fitness between genotype, temperature, and sexual selection intensity to influence allele frequency dynamics in *6Pgdh* in *R. robini*. Our findings suggest that the interplay between the abiotic environmental factors (temperature) and social environment (sex ratio) may contribute to the maintenance of polymorphism in this system, though further investigation is needed to clarify the underlying mechanisms. While we were unable to detect clear differences in reproductive fitness between genotypes at different temperatures, our results encourage a more comprehensive approach to studying context dependent selection, where the combined effects of different environmental and social factors are considered. Future research focusing on long‐term experiments, survival‐related fitness components, and fluctuating environmental conditions could provide deeper insight into the evolutionary dynamics shaping genetic diversity in natural populations.

## Author Contributions

**Pranav Unnikrishnan:** conceptualization (equal), formal analysis (lead), investigation (equal), methodology (equal), visualization (lead), writing – original draft (lead), writing – review and editing (equal). **Anna Spaeth:** data curation (supporting), investigation (supporting), methodology (supporting), project administration (supporting). **Magdalena Trojańska:** data curation (supporting), methodology (supporting), project administration (supporting), writing – review and editing (supporting). **Wiesław Babik:** conceptualization (equal), data curation (supporting), formal analysis (supporting), investigation (supporting), methodology (supporting), project administration (supporting), supervision (equal), validation (equal), visualization (supporting), writing – original draft (supporting), writing – review and editing (equal). **Agata Plesnar‐Bielak:** conceptualization (lead), data curation (equal), formal analysis (equal), funding acquisition (lead), investigation (equal), methodology (equal), project administration (equal), supervision (lead), validation (equal), visualization (supporting), writing – original draft (supporting), writing – review and editing (lead).

## Funding

This work was supported by Narodowe Centrum Nauki, UMO‐2019/33/B/NZ8/02442.

## Conflicts of Interest

The authors declare no conflicts of interest.

## Data Availability

All the required data are uploaded to the dryad repository: https://doi.org/10.5061/dryad.crjdfn3jp. The sequences used for genotyping, prmiers and probes are uploaded as Appendix A.

## Appendix A

### Primer, Probe, and Sequence Information for the *6Pgdh* TaqMan SNP Genotyping Assay

**Table:**

Component	Sequence (5′ → 3′)	Dye/quencher
Forward primer	CAGGACCTGAAAATACCAGATACGA	—
Reverse primer	AGACAATCTTAGAGGCATACAAAGCC	—
Probe (S allele)	CACACGTACCATTCGGA	VIC/MGB‐NFQ
Probe (F allele)	CACACGTACCCTTCGGA	FAM/MGB‐NFQ

The forward and reverse primers flank the missense S/F SNP in the *6Pgdh* coding region. Allele discrimination is achieved using two fluorogenic minor‐groove‐binder (MGB) probes differing at the SNP position, labeled with VIC (S allele) and FAM (F allele). All oligonucleotides were supplied as part of a Custom TaqMan SNP Genotyping Assay (Thermo Fisher Scientific).

### Sequence Context Used for Assay Design

The following *6Pgdh* sequence fragment shows the region surrounding the S/F polymorphism, with ambiguous bases corresponding to the SNP position (this is the sequence that was provided to Thermo Fisher for assay design):‐ AAGAAGAACGAGTGCAGGCTAGCAAAATATTAGCAGGACCTGAAAATACCAGATACGAAGGNGATAAACAGGCTTTCATTGAGGANATCCGAAKGGTACGTGTGAACAAAACTATTTTCNANTTATCACATTTTAAATAATTTGTTGTCTTATCCCTCTNAGGCTTTGTATGCCTCTAAGATTGTCT.
